# What social media analyses can tell us about Ghanaian women's concerns during pregnancy

**DOI:** 10.3389/fdgth.2025.1479392

**Published:** 2025-02-13

**Authors:** Martina Anto-Ocrah, Tori Valachovic, Joseph W. Lanning, Ali Ghanem, Claire Couturier, Celestin Hakizimana, Celestin Niyomugabo, Nabeeha Jabir Affan, Hemika Vempalli, Ruth Sally Kodam

**Affiliations:** ^1^ Division of General Internal Medicine, University of Pittsburgh School of Medicine, Pittsburgh, PA, United States; ^2^ Department of Epidemiology, University of Pittsburgh School of Public Health, Pittsburgh, PA, United States; ^3^ University of Rochester School of Medicine, Rochester, NY, United States; ^4^ Sustainable Development Practice, School for International Training Graduate Institute, Brattleboro, VT, United States; ^5^ Department of Neurology, UT Southwestern Medical Center, Dallas, TX, United States; ^6^ University of Pittsburgh School of Health and Rehabilitation Sciences, Pittsburgh, PA, United States; ^7^ Peace Corps, Conakry, Guinea; ^8^ VONSUNG, Kigali, Rwanda; ^9^ MidWife Sally Organization, Dawhenya, Ghana

**Keywords:** pregnancy, Ghana, Africa, social media, machine learning, Facebook

## Abstract

**Introduction:**

Social media platforms are used by over 4.9 billion people for networking and community building, as well as for healthcare information seeking and decision-making. Most studies investigating the utilization of social media during pregnancy have focused on high-income countries, despite the surge in social media utilization globally. In this study, we analyzed how pregnant women in Ghana, West Africa, utilize Facebook to inform decision-making on their most salient pregnancy concerns.

**Methods:**

We utilized machine learning techniques (Web Scraping and Latent Dirichlet Allocation) to mine and analyze posts from the Ghana-based MidWife Sally Pregnancy School Facebook group between August 16, 2020 and April 29, 2023. Posts were extracted, cleaned, and analyzed using Gensim python library. Topics were generated based on their probabilities and relevance to the study goal.

**Results:**

A total of 3,328 posts were extracted and 3,322 were analyzed after removing 6 empty posts. Seven major topics with listed subtopics were identified: Pregnant (693 posts): personal physiological changes, exercise during pregnancy, medication (e.g., anti-malarials, pain killers) Delivery (367): emergency delivery, vaginal/caesarean birthing, breastmilk production, exercise during pregnancy Pain (350): location of pain and pain relief modalities (e.g., exercise, medication, sleep) Breastfeeding (248): delivery, emergency service, milk production Water (174): cold water consumption, infant feeding (e.g., gripe water, constipation, formula) Sleeping (165): discomfort, sleeping positions, exercise to induce sleep, sleep as a natural analgesic Antenatal (124): fetal growth, progress, hospital selection Of note, content from “Pregnant”, “Delivery” and “Sleeping” included mentions of depression, while “Breastfeeding” highlighted cultural approaches to increasing milk production. The sentiment analysis showed that 43.4% of the responses were neutral and primarily focused on seeking information. Negative sentiments, which were more distressing, comprised 46.4% of the responses, while positive sentiments, had a celebratory tone and represented 10.2% of the data.

**Conclusion:**

Social media analysis, previously employed in high income settings, can provide impactful, granular snapshots of pregnant people's concerns in the African region, which could be used to inform social media interventions aimed at filling educational gaps in antenatal care for those without adequate healthcare access.

## Introduction

As internet access expands worldwide, people increasingly rely on online platforms to find health information quickly and connect with others who share similar experiences or identities (1, 2). This trend has sparked interest among digital public health researchers who aim to analyze user data to better grasp their needs and develop effective interventions. Of particular interest to women's health researchers is women's social media and internet utilization during pregnancy due to both the inherently life-changing nature of pregnancy as well as increasing concern around maternal health in the peripartum period and infant health in the postpartum period.

There have been multiple studies of women in high income countries, such as the United States, United Kingdom, Canada, and Australia (3–5), investigating their social media and internet use during pregnancy. These studies have revealed that utilization of online sites for health information during pregnancy is largely ubiquitous (4). Findings from these studies show that, overall, pregnant women in these settings find online resources to be helpful, trustworthy and a desirable source of social support, especially for those with barriers to care (5–7). Notably, there have been studies of midwife-moderated social media pages that have been very promising in terms of facilitating patient education and creating therapeutic relationships (8, 9). As a result of these studies, many authors have postulated social media as a viable tool for improving accessibility to health education during pregnancy for patients. Limited, however, is research on social media utilization amongst pregnant women in resource-limited settings, such as Ghana, West Africa, where we are seeing a global surge in internet access and social media utilization.

As of April of 2023, quarter-on-quarter change in reported Facebook advertisement reach showed an increase of 57 million viewers in Africa alone (10). This is a 26.8% increase, which compared to the 8.5% increase in quarter-on-quarter change in ad reach seen in the Americas during the same time period, is a compelling example of the burgeoning growth of social media use in the African region (10). In Ghana specifically, the percentage of the population using the internet has increased dramatically since 2017, from a reported rate of 38% to then 68% in 2021 (11). Four in ten internet users in Ghana report using social media to assess health information (12), and multiple studies have explored how internet users across various African countries interact with social media sites for health information, including: adolescent sexual health (13), HIV/AIDs (14), and mental health interventions (15).

However, no studies have, to our knowledge, focused on the use of social media amongst women in the African region who are, or trying to become, pregnant to distill their pregnancy-related concerns. This gap is especially concerning, given that the African region accounts for 70% of the world's maternal deaths (16). Understanding the types of information these women are seeking from social media could help in intervention development.

The purpose of this study was to use machine learning and natural learning processing techniques to analyze posts on the *Enjoy Your Pregnancy* Facebook Page, a social media platform designed to provide tailored educational content to followers who are actively pregnant or trying to become pregnant, to better understand women's needs during their pregnancy journeys.

## Methods

### Data source

The Midwife Sally Pregnancy School (“Midwife Sally”) a Ghanaian non-governmental organization (https://www.facebook.com/MidwifeSallygh/), is a social-media platform that offers antenatal and post-partum support to pregnant women. With an active community of 233,000 Facebook followers (as of October 2023), Midwife Sally offers pregnancy educational videos and written content to empower women on their pregnancy journeys (17). Members are encouraged to share their pregnancy experiences and engage with midwife providers and each other by posting pregnancy-related questions or concerns, which are addressed by providers in real time on the platform. Most of the Midwife Sally public page followers with geolocation data reside in Ghana (86.0%, *n* = 71,768), 8.5% (*n* = 7,165) are in Nigeria, 4.0% (*n* = 3,353) are in Zambia, and 1.9% (*n* = 1,622) reside in Kenya.

The *Enjoy your Pregnancy* Facebook page, which was the source of the data extraction for this study, is a private, subscription-based subsidiary of the Midwife Sally Pregnancy School for members who desire more tailored pregnancy educational content. Participants are given access to asynchronous videos and educational content that span a variety of pregnancy-related topics as well as live weekly classes. Topics with the highest levels of engagement at the time of the study included: Wound care after cesarian section, Nutrition During pregnancy, and Danger Signs in newborns (Supplementary Table 1).

### Patient and public involvement

The study was conceptualized by final author Ruth Sally Kodam (RSK), founder of MidWife Sally and all affiliated initiatives, including the pregnancy school. There was no direct involvement of participants or other community members in the design, conduct, reporting, or dissemination plans of the research.

### Data extraction

**Figure 1:**
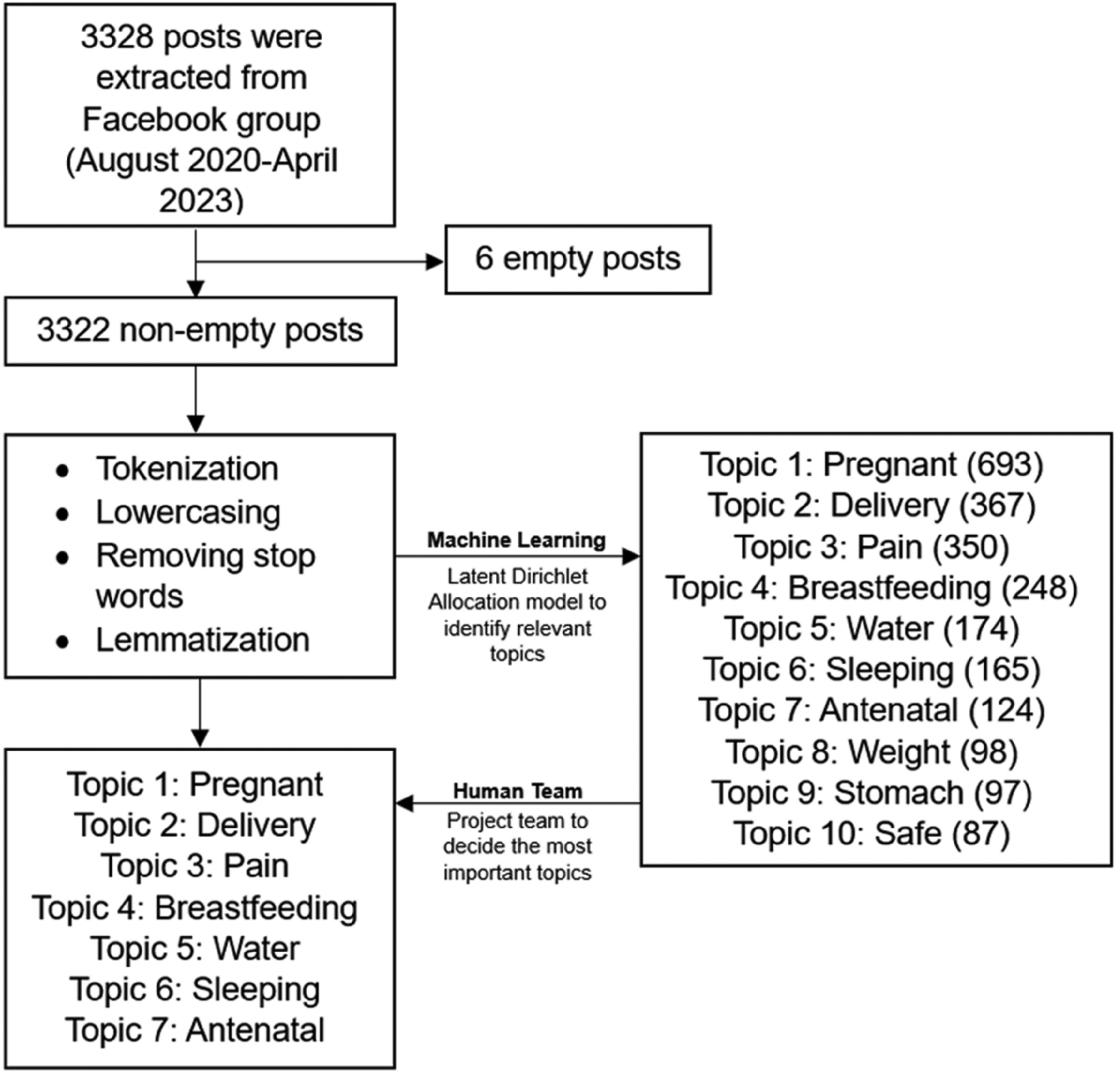
Flowchart of Facebook post data analysis. Numbers in parenthesis (*n*) represent the number of posts that contain the specified topic.

We used python programming language libraries (pandas, BeautifulSoup and selenium) and web scraping to extract posts made between the start date of the page, August 16, 2020, and April 29, 2023 on the *Enjoy Your Pregnancy* Facebook page (Figure 1). The study was conceptualized by final author Ruth Sally Kodam (RSK), founder of MidWife Sally and all affiliated initiatives, including the pregnancy school. We followed the ethical guidelines set forth by the Association of Internet Researchers (18) which emphasizes respect for user privacy and dignity when utilizing social media data. Ms Kodam was involved in all aspects of the study from conceptualization to data extraction, analyses, interpretation, and manuscript drafting and finalization to ensure the utmost respect of the data was being upheld.

### Data preprocessing

It is very common to see multiple comments in these groups in one post, not only responding but asking follow-up or new questions. We analyzed all comments and responses in aggregate to provide a fuller picture of the women's pregnancy concerns.

To prepare the data for topic identification, we removed all empty posts, then further cleaned the remaining data using the following approaches:

1. **Tokenization**: We split texts into individual words. This step helps in breaking down the text into meaningful units for further analysis.
2. **Lowercasing**: Converted all text to lowercase so words are treated consistently.
3. **Removing stop words**: Stop words are common words like “the,” “and,” “is,” which occur frequently in a language but often do not carry significant meaning. Removing them can reduce noise and improve processing efficiency.
4. **Lemmatization**: This technique reduces words to their base or root form. It considers the context and converts words to their dictionary form (lemma).

### Topic identification (19–21)

We applied Latent Dirichlet Allocation (LDA), an unsupervised topic modeling approach, to the posts to identify distinct topics within the data. LDA model was performed using Gensim python library to generate the topics for the study. The goal of topic identification is to uncover the underlying themes in a collection of documents, and the coherence score is a key measure to assess how interpretable and meaningful these topics are.

A higher coherence score indicates that the words within a topic are more likely to co-occur and make sense together, leading to more meaningful topics. LDA was chosen as the most effective model for topic modeling due to its ability to produce semantically coherent and interpretable topics, as demonstrated by its highest coherence score of 0.7368 (Table 1). This score indicates that the words within each topic are highly related to one another, making the topics generated by LDA meaningful and easy to interpret.

**Table 1:** Coherence score for different models tested for topic identification.

Model	Coherence score
LDA (Latent Dirichlet Allocation) model	0.7368461780809704
DTM (Document-Term Matrix)	0.4627887678448415
HDP (Hierarchical Dirichlet Process)	0.697539548850166
Latent Semantic Analysis (LSA)	0.4289135433961876

### Disaggregation by topics

After identifying the topics, we aggregated posts that contained specific topics together, and analyzed the data topic by topic.

### Topic analysis workflow

1. Bag of words

Bag of words is a natural language processing (NLP) technique that is used to represent a text document into numerical form by considering the occurrence of words in the given document. To create bag of words, “CountVectorizer” which is a python built-in function from “sklearn” library was used. “CountVectorizer” has different parameters including “ngram_range” which specifies the range of words to be in our bag of words, there is also “min_df” which defines the minimum number of frequencies a word must have to be in bag of words and “max_df” which excludes the words with exceeds the frequency of the specified number.

Bag of words forms a new dataframe and each column is represented by each word or combinations of words specified in n-grams.

2. N-Grams

N-gram specifies the combination of words to create the bag of words. In “CountVectorizer” we create N-Grams using the ngram_range (a,a) and we specify the number of words in the parenthesis. In our study, we specified n-grams to be (3,3), which means that we want our key words to be a combination of three words (trigrams). After specifying n-grams (3,3) for each topic, dataframe contained the combined and its frequency were created.

3. Text combinations

Some text generated had similar meanings, so, in consultation with RSK's clinical expertise, we manually combined and selected groups of the words that were clinically meaningful, reduced redundancy, and jargon. For Example for the first post regarding breastfeeding in Table 2 below, the following 4 trigrams (bags of words) were produced:

- meals eat increase
- eat increase breast
- increase breast milk
- breast milk production

**Table 2:** Example of text combinations for trigrams.

Original text	Processed text (stop-words removed)
What meals can one eat to increase breast milk production	Meals eat increase breast milk production
Dear midwifes and mums pls is it true that as a breastfeeding mum always wearing a bra and even sleeping with it decreases breast milk production? and also is it true drinking cold water and drinks slows down milk production?	Dear midwifes true breastfeed always wear bra sleep decrease breast milk production true drink cold water drink slow milk production

With RSK's guidance, the group decided that the trigram “breast milk production” would make the most clinical sense to include. This trigram was also meaningfully captured in the 2nd post.

4. Visualization

Results of bag of words and n-grams were converted into a dataframe, which has columns of words combination and frequency for each. A bar chart was used to represent the data on the graph using matplotlib python library.

5. Sentiment analysis

Sentiment analysis, also referred to as opinion mining, is an approach to natural language processing that identifies the emotional tone behind a body of text. Sentiment analysis is the process of analyzing digital text to determine if the emotional tone of the message is positive, negative, or neutral.

Below are some key definitions for the sentiment analysis:

**Polarity:** Polarity is the measure of the overall combination of the positive and negative emotions in a sentence.
**Positive polarity:** It indicates a favorable or positive sentiment. Text with positive polarity often expresses satisfaction, joy, admiration, or excitement.
**Negative polarity:** It indicates an unfavorable or negative sentiment. Text with negative polarity often expresses dissatisfaction, disappointment, anger, or frustration.
**Neutral polarity:** It indicates a lack of strong sentiment or an unbiased tone. Text with neutral polarity typically does not express strong emotions.

In our study, we apply sentiment to each post to understand the emotions of each behind each post.

To apply the sentiment, VADER [Valence Aware Dictionary and Sentiment Reasoner (22)] was used. The VADER model assigns sentiment scores to words based on their intensity and polarity, considering the context and grammatical rules of the text. As a result, a web app was designed to display the sentiment of posts.

## Results

**Figure 2:**
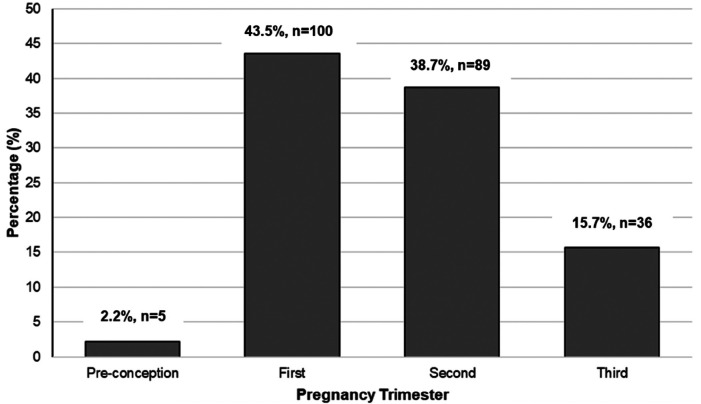
Pregnancy trimester status of members in the midwife sally *enjoy your pregnancy* Facebook page who provided data about their pregnancies (*n* = 230).

There were 481 members subscribed to the Midwife Sally *Enjoy your Pregnancy* page at the time of data collection. Among the respondents who shared information about their pregnancies (*n* = 230), most were in either their first (43.5%) or second trimester (38.7%) (Figure 2).

Out of a total of 3,328 posts extracted from the Facebook page, 3,322 were analyzed after excluding 6 empty posts. Utilizing LDA modeling on the dataset, we excluded an additional 991 posts containing topics unrelated to the research question, such as advertisements for live educational sessions and reminders for subscription fee payments. From the remaining 2,403 posts, we identified 7 overarching topics that best captured the most frequently discussed and relevant ideas posted by page participants: Pregnant, Delivery, Pain, Breastfeeding, Water, Sleeping and Antenatal. Extracting three-word phrases associated with each major topic provided a more detailed understanding of which specific aspects were most significant to participants. The sentiment analyses for each topic can be found in the Supplementary Materials.

### Pregnant

**Figure 3:**
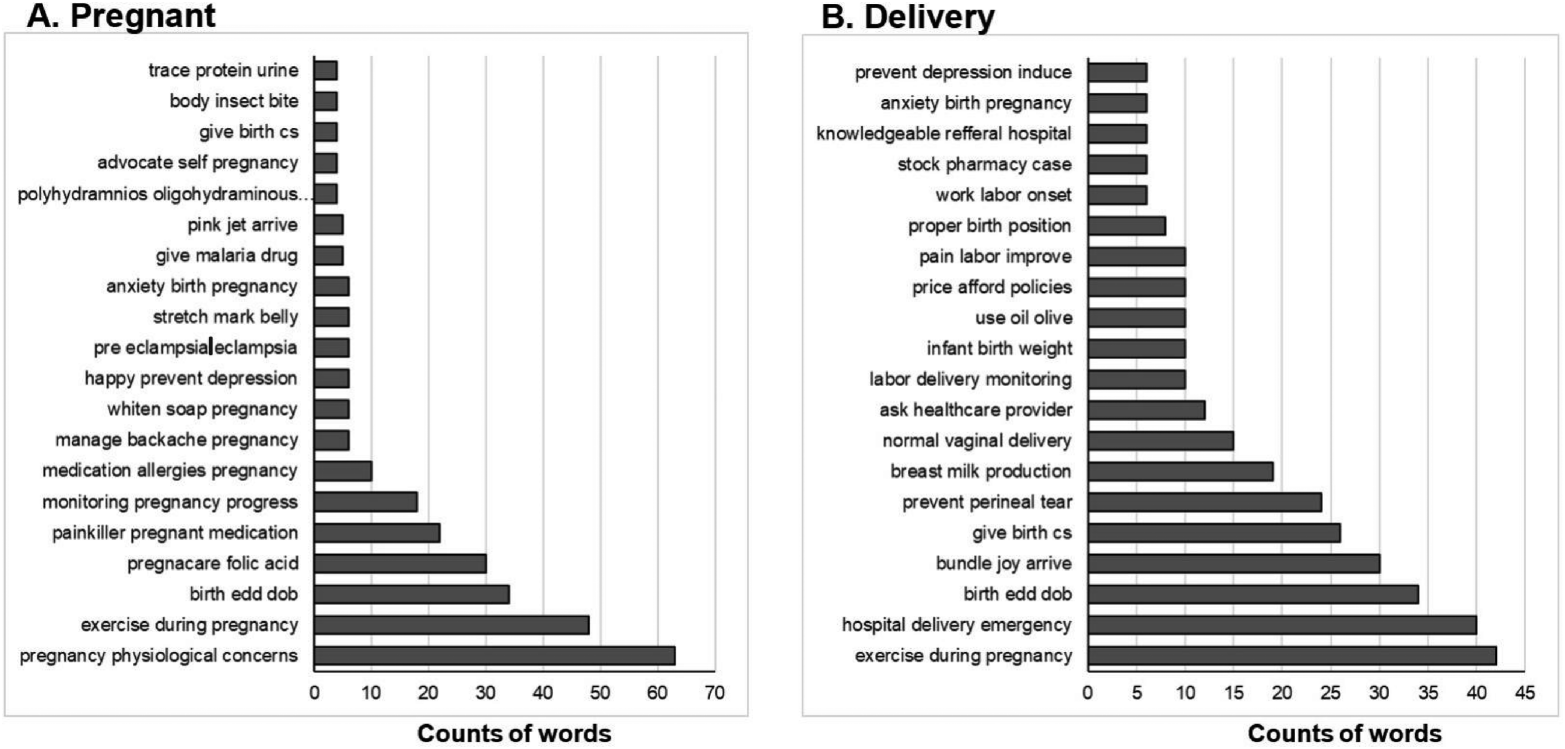
**(A)** Most frequent phrases associated with topic of “Pregnant”. Total *n* = 693 posts. **(B)** Most frequent phrases associated with the topic of “Delivery”. Total *n* = 367 posts.

The topic identified as “Pregnant” was present in 693 posts from the page. It was most referenced in relation to physiologic concerns (*n* = 63), exercise during pregnancy (*n* = 48), estimated due dates or birth dates (*n* = 34), folic acid supplementation (*n* = 30), painkiller use in pregnancy (*n* = 18) and monitoring pregnancy progress (*n* = 18, Figure 3A). Additionally, topics such as mental health (depression, *n* = 6; anxiety, *n* = 6) and self-advocacy (*n* = 4) also surfaced in relation to the topic.

Posts that captured the essence of the topic included:

“morning midwives am 31weeks pregnant and I feel a lot of pressure in my vagina pelvic and waist pains the same time and at the same time having running stomach both day and night am really suffering what might be the cause”

“morning my fellow ladies. How do I regain my libido as I am pregnant it's really worrying me”

“i am 14 weeks now but started taking folic acid the very day I got to know I was pregnant on the day of my antenatal i was given vitafol and vitamin c and still on the folic acid want to know what does the vitamin c and vitafol do”

“am 17 weeks I can eat pawpaw pineapple these are my favourite fruits but I heard pregnant woman cant eat pineapple pawpaw especially”

“Exercise during pregnancy exercising during pregnancy can make your labor and delivery easier make it a practice and ask your health care provider which one is convenient for you there are simply exercises that a pregnant woman can do example walking”

“ what is the sex positions for pregnant woman” “is it normal to be very depressed when pregnant I cry almost everyday and I stay alone I’m in my early stage”

### Delivery

“Delivery” was the second most identified topic with mention in 367 posts. Amongst the most frequent phrases associated with this topic were exercise during pregnancy (*n* = 42), emergency hospital delivery (*n* = 40), estimated due date or birth date (*n* = 34), and arrival of the baby (*n* = 30, Figure 3B). Multiple posts contained references to delivery methods such as caesarian section (*n* = 26) and normal vaginal delivery (*n* = 15), as well as other aspects of the delivery such as preventing perineal tears (*n* = 24), labor delivery monitoring (*n* = 10) and proper birthing position (*n* = 8). A substantial number of posts also mentioned breast milk production (*n* = 24) in relation to “Delivery.” Additionally, posts included references to seeking assistance such as asking a healthcare provider (*n* = 12) and knowledgeable referral hospitals (*n* = 6). Notably, mention of mental health co-occurs with the topic of “Delivery” as there are posts about anxiety related to birth (*n* = 6) and depression prevention (*n* = 6). Many also returned to share their birth stories and successful deliveries, thanking the Facebook platform for the teachings.

Posts on this topic included:

“ madam midwife by gods grace i had a delivery via cs on account of gestational hypertension and previous cs at week 38 1 my actual edd was 1st august god bless you for your educational programs and lessons your advice and encouragement made me go for”

“ midwives and mums can a pregnant woman tells her doctor she wants elective cs even though everything is for vaginal delivery”

“ midwives a first time mum i delivered on the 2nd june baby will be 6 weeks on thursday i delivered through vaginal delivery and i was just given epistemology stopped bleeding on my 5th week after birth however just this friday i realized i have stains”

“heloo midwives am sad sometimes not because of the pregnancy but i feel i dont have anybody …after delivery my mommy has passed away and my husby mummy is not close at all so it obvious she cannot come hmmmm i feel a bit left out ”

“alhamdulilah for a delivery …thanks to u all for the teachings”

“Hello abusuafo, being a first time mom isn't easy…the struggle I go through everyday with my baby hmm..pardon me I couldn't post my birth story been very depressed lately… But by God's grace my baby and I are fine.im really grateful to all the Midwives and everyone here. God bless you all…”

### Pain

**Figure 4:**
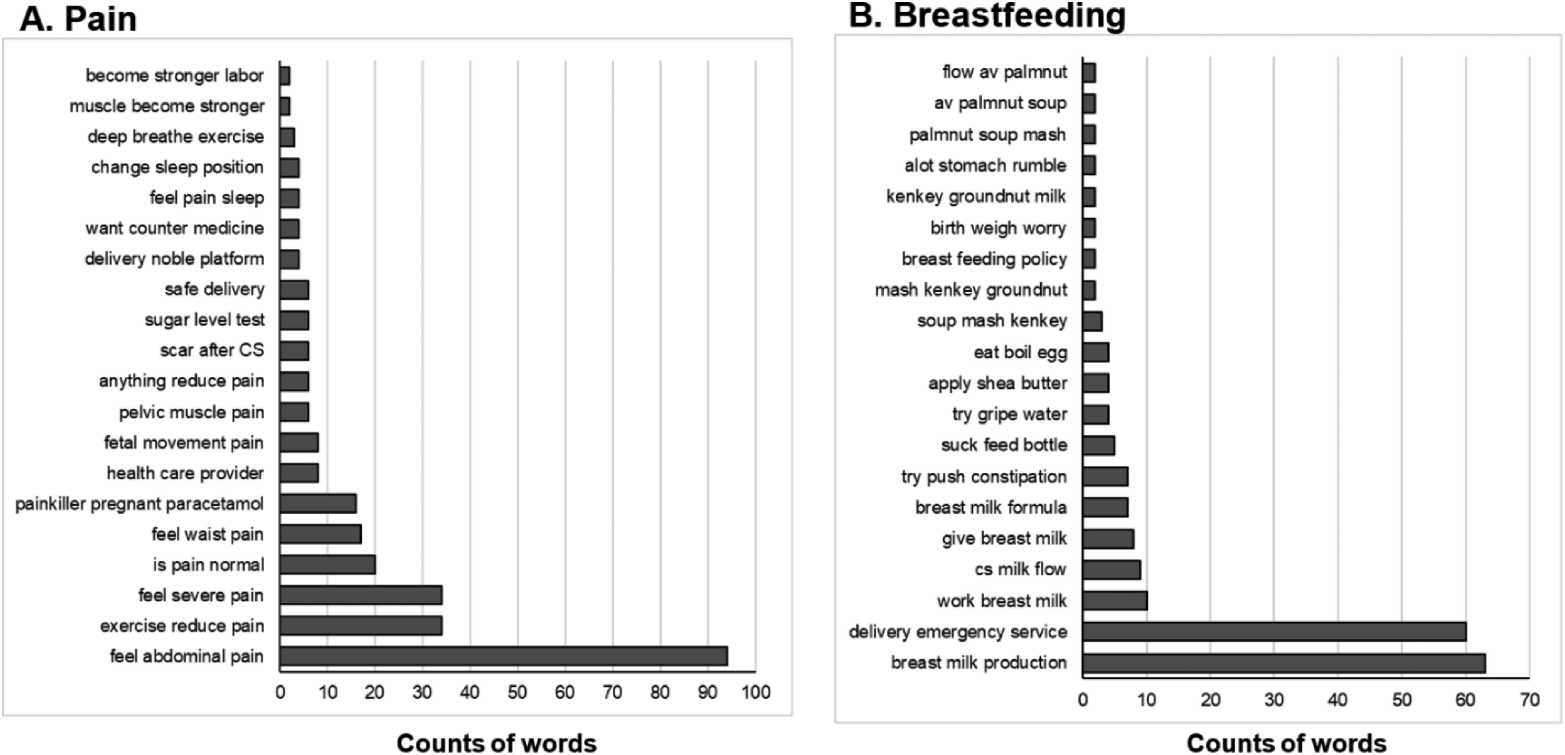
**(A)** Most frequent phrases associated with topic of “Pain”. Total *n* = 350 posts. **(B)** Most frequent phrases associated with the topic of “Breastfeeding”. Total *n* = 248 posts.

The third most frequently occurring topic was “Pain” found in 350 posts (Figure 4A). By far the most common association with “Pain” was the experience of feeling abdominal pain (*n* = 94). Other similar phrases included feeling severe pain (*n* = 34), waist pain (*n* = 17), fetal movement pain (*n* = 8), and pelvic muscle pain (*n* = 6). Additionally, there were multiple phrases referencing if there is anything for pain reduction (*n* = 6), and actual methods for alleviation, most prevalent being exercise to reduce pain (*n* = 34), but also including painkiller paracetamol during pregnancy (*n* = 16), over the counter medication (*n* = 4), changing sleeping position (*n* = 4), and deep breathe exercise (*n* = 3).

Posts that captured the essence of the topic included:

“ family im 31weeks along and have been feeling severe pain in my pubic pelvic bones such that i cannot even walk properly or lift my feet up babys movements are still normal should i be worried what does this mean”

“midwifes abeg oo is toothache accompanied with headache common in third trimester im 30 weeks can i take painkiller i need”

“hi im 28 w 2d i have been experiencing this dull but pressing pain in my lower abdomen for 2 days now it hurts a little in my vagina is this normal”

“afternoon mummies and midwives im currently 31wks started feeling pain at my right hip and waist baby hasnt been so active today i feel baby position is stressing it and causing me pain what should i do pain sometimes hitting under my right breast ”

### Breastfeeding

“Breastfeeding” was identified as an overarching topic in 248 posts from the page. It was most associated with concerns of breast milk production (*n* = 63) and emergency delivery services (*n* = 60, Figure 4B). Other associated phrases related to improving breast milk production or flow, including after having a caesarian section (*n* = 9) or utilizing adjunct methods such as applying shea butter (*n* = 4), consuming kenkey (*n* = 7), groundnut *n* = 4), and palmnut (*n* = 6). Additionally, there were concerns regarding infant wellbeing in relation to breastfeeding, such as constipation (*n* = 7), using gripe water (*n* = 4) and birth weight (*n* = 2).

Posts on this topic included:

“ mums and midwives when is the bleeding suppose to stop after csi has cs and is six weeks now but am still bleeding and i feel pain at my back when breastfeeding how do i go about it a first time mum”

“ midwives how do i care for my nipples for breastfeeding it seems my nipples are blocked”

“morning my fam and wonderful midwives can anyone recommend 1 month family planning for a breastfeeding mum”

“afternoon can breastfeeding mothers take sobolo”

“midwife sally i once saw a post of a list of meals breastfeeding mums can eat to boost their milk supply can you post it on here?”

### Water

**Figure 5:**
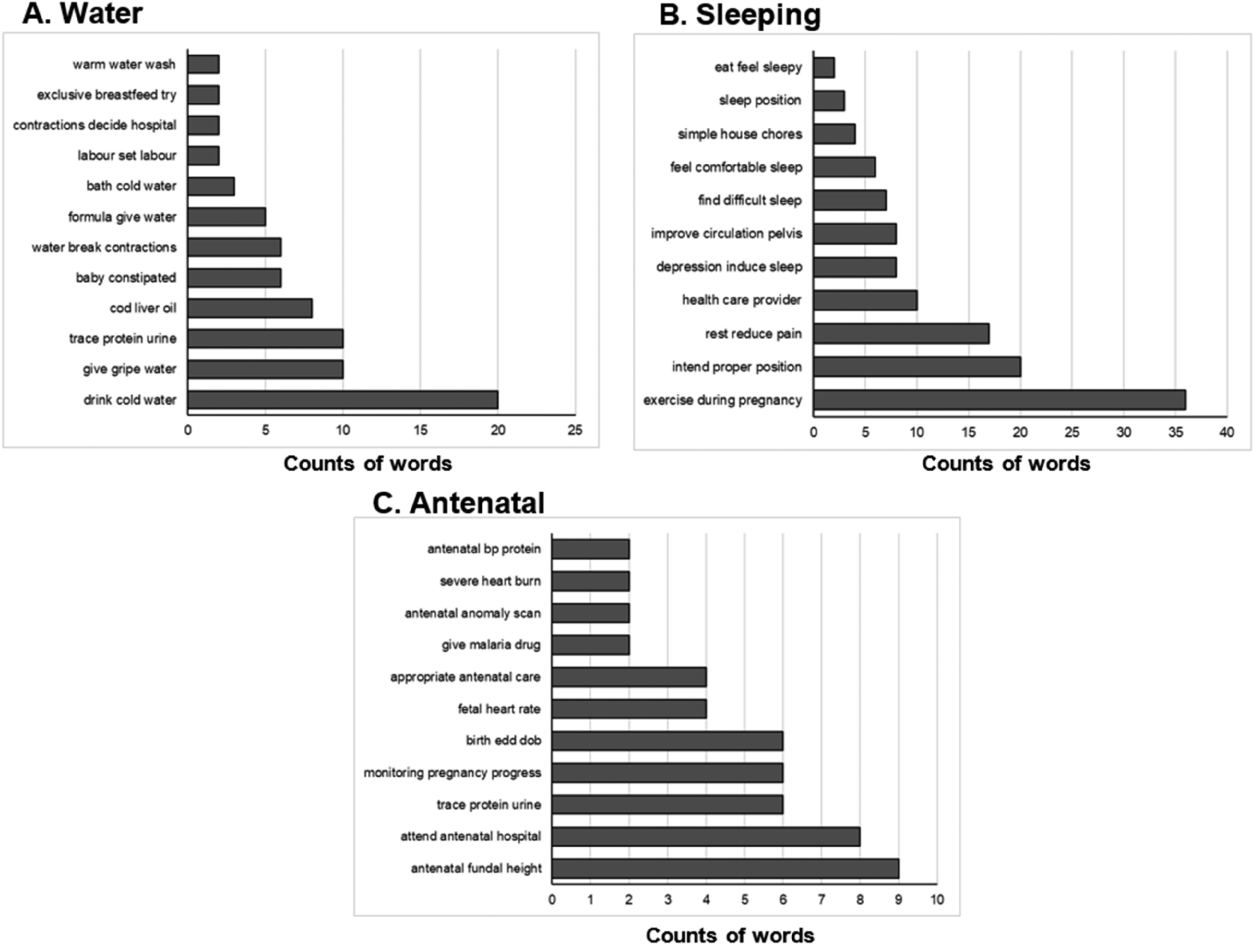
**(A)** Most frequent phrases associated with topic of “Water”. Total *n* = 174 posts. **(B)** Most frequent phrases associated with the topic of “Sleeping”. Total *n* = 165 posts. **(C)** Most frequent phrases associated with the topic of “Antenatal”. Total *n* = 124 posts.

Another prevalent topic in the Facebook group was “Water”, identified in 174 posts. Most commonly this topic was associated with drinking cold water (*n* = 20, Figure 5A). There were also many phrases centered on infant needs such as giving gripe water (*n* = 10), constipation (*n* = 6), giving water with formula (*n* = 5), trying exclusive breastfeeding (*n* = 2) and warm water washes (*n* = 2). “Water” was also associated with topics directly related to pregnancy such as health concerns around proteinuria (*n* = 10), water breaking contractions (*n* = 6), onset of labor (*n* = 2) and deciding to go to the hospital with contractions (*n* = 2). Additionally, cod liver oil was found in relation to the overarching topic (*n* = 8).

Posts that captured the essence of the topic included:

“morning midwives is it that after eight months of pregnancy one is not supposed to bath cold water”

“ a friend lost her pregnancy at 25 weeks due to her water leaking what are the possible causes and how can it be prevented again how do you detect your water is leaking ”

“afternoon midwives am worried me theres always trace of protein in my urine but my bp is normal i take a lot of water too 28wks 6days protein trace ”

“ midwives im almost 12 weeks i cant drink water there is this bitter taste on my tongue and it makes it difficult for me to drink water i have to take the water in sips or else i would have to turn it all up even with that im not sure i can drink two”

### Sleeping

165 posts were coded to have an overall topic of “Sleeping”. This topic was most often associated with the phrases exercise during pregnancy (*n* = 36), proper position (*n* = 20), and rest to reduce pain (*n* = 17, Figure 5B). Reference to seeking a health care provider (*n* = 10), mental health concerns of depression inducing sleep (*n* = 8) and improving pelvic circulation (*n* = 8) were also relatively common. Other phrases related to difficulty (*n* = 7) or comfort (*n* = 6) with sleep, sleep position (*n* = 3), completing simple house chores (*n* = 4) and eating related to feeling sleepy (*n* = 2) were identified, but not frequently referenced.

Relevant posts included:

“ midwives am 37wks 2days gone i now find it difficult with my sleeping positions husby has to me up and down from bed there is also so much pressure in my abdomen when baby kicks when i walk for some time i feel some pressure either in my left or right”

“morning midwives and mothers i have been having this painless but very uncomfortable contraction the intervals in which it comes is very short making sleeping difficult for me could it be labouri will be 40weeks tomorrow”

“ at what stage must i start sleeping on my left side im very early in this journey”

“in third trimester but cant sleep at night will sit down aaa till 12am then try to sleep not sleep biaaa”

“ everyone my 8 weeks old baby doesnt sleep nowadays not even once during the day instead he sucks a lot … he can suck breast milk the whole day and still cry for more what seems to be the problem first time mum”

### Antenatal

The topic of “Antenatal” was identified in 124 posts (Figure 5C). Most of the associated phrases were in relation to experiences or terms utilized in medical settings such as fundal height (*n* = 9), attending an antenatal hospital (*n* = 8), monitoring pregnancy progress (*n* = 6), estimated delivery date or birth date (*n* = 6), fetal heart rate (*n* = 4), and anomaly scan (*n* = 2). Others were related to health concerns in the antenatal period such as trace proteinuria (*n* = 6) or blood pressure protein (*n* = 2), malaria treatment (*n* = 2), and severe heart burn (*n* = 2).

Posts included:

“I'm a first time mum and 5 months pregnant…I went for antenatal yesterday and as usual I went with questions but I'm not satisfied with the answers I was given”

“morning midwives i went for antenatal yesterday and these were the findings bp11080 protein trace sugar negative fundal height 23 fetal heart rate 139 what am worried about is the trace of protein in the urine…what can i do to eliminate”

“ midwifes and mummies am 35 weeks today and went to the clinic for antenatal only for my doctor to tell me she will deliver baby at 38 weeks because my bp hasnt been stable i asked her if its cs but she said no unless babys weight is above 4kg”

“morning family i need a recommendation on a hospital … for antenatal care and delivery services ”

### Sentiment analysis

**Figure 6:**
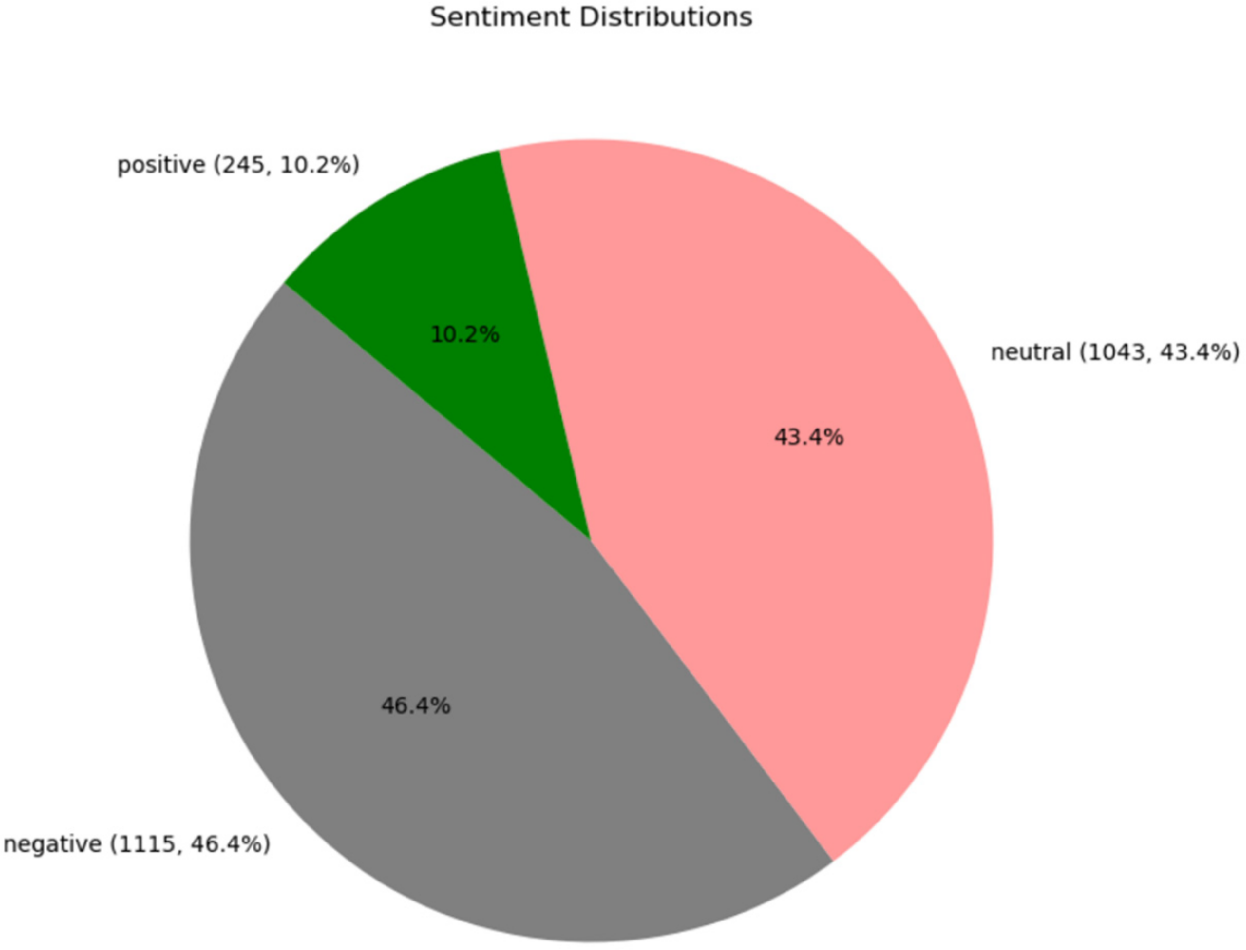
Distribution of sentiments (*n* = 2,403 posts).

The sentiment analysis showed that 43.4% of the responses were neutral and primarily focused on seeking information. Negative sentiments, which were more distressing, comprised 46.4% of the responses, while positive sentiments, which had a celebratory tone, represented 10.2% of the data (Figure 6).

Examples of the sentiments and their classifications included:

**Neutral sentiments:**

1. “please i heard there are 4 types of contractions can u teach me on it and also when do i know when labour sets in and when to go to the hospital pls. ready to learn as a first time mum”
2. “good afternoon midwives! please; 1. in which week should i be given malaria drug 2. in which week should tetanus be administered to me?”
3. “hi midwives, goodevening pls can a pregnant woman use powder on her armpit and [vagina]??”
4. “Hello evening mid wives and mummies, please when is it Ideal to start weight loss after giving birth and breastfeeding too?”

**Negative sentiments:**

1. “So yesterday, I had an electrician come over to help me fix a curtain bar. I was carrying my 7 week old baby and showing him how I wanted it done. Because its a new place, i had things scattered and didn't see the wires he used for the connection on the floor. I stepped on a life wire and got an ‘electric shock’. Infact it wasn't easy and had it not been God, I don't know what would have happened to my baby and I. Mummies, please let us be vigilant in all we do. For me, I've learnt my lesson. #safety first.”
2. “Good day midwives at what weeks is the baby supposed to kick or move iam 22 weeks today and i felt a little movement in my abdomen and the left side of my stomach but it is not consistent.i need your advice pls. first time mum”
3. “what do we do about body of our during the last trimester, am 40 weeks and the past one week my armpit smells like onion. i have used lime, my regular roll on and perfume, and joy ointment but still i can smell it.”
4. “good day midwives. i have been down with cold and cough the whole week. visited the hospital but the dr said she wouldn't give me any drugs except vitamin c and lozenges. she does not prescribe antibiotics or drugs. she said it is best since I'm 33 weeks. she said i shd try natural like lemon and honey with tea. I'm struggling cos i vomit when i take the lemon honey tea. what else can i do pls.”
5. “Hello midwives, Please is it advisable to prescribe and use quinine on a toddler [who is] 2 years and 6 months old. I carried my son to the hospital because he was burning up and they told me he had malaria. Immidiately they fixed drips with quinine on it and made him stay at the hospital for more than 4 days, I even had to disagree that I would not let him…stay more than that, because the mosquito there was too much and his temperature was going up and down and they discharged him and said he was fine… then gave me extra 3 bottles of quinine and some other drugs, that I should give him the drugs till 7 days. But up till now, he is still burning up and having blurred vision and also complaining of stomach ache. I read online that children under 16 years of age should not be given quinine that it might lead to kidney failure or death…I don’t even know what to do, am just here confused”

**Positive sentiments**

1. “good evening mummies. by the grace of god my blue jet arrived exactly a week ago. birth story will follow soon.”
2. “good evening ms sally, please i am a first time pregnant woman and my husband mentioned that i should be taking abeduru blending with fresh orange juice to drink. and that it's healthy. please what's your take on this? thanks a lot”
3. “hello, i want to tell all pregnant women on this platform not to joke with the turkey berry juice. its very effective. i can’t believe my [hemoglobin] which was 8.6 has risen to 10.1 just with a week oo. i drink it as my water.i take my medications as well… im very happy about this [hemoglobin improvement]. i will continue to drink till i deliver. i’m 37 weeks. thanks”
4. “alhamdulilah for a safe delivery. pls help me thank midwife … @; prampram polyclinic for their exceptional services. thanks to u all for the teachings.”
5. “To God be all the glory, great things He has done! My baby girl has arrived after 20 hours of induction and painful labor, but God showed himself strong. A big [thanks] to all the midwives on this platform and all mum's who encouraged me wen I was going for the induction. I'm really grateful… And I learnt a lot on this platform even though I was a quiet member. God bless you madam Sally for all you're doing”

## Discussion

Utilizing machine learning techniques to directly analyze posts from a private Facebook group for pregnant women in Ghana, we were able to determine with significant granularity the topics of interest generated by these women during their pregnancy journeys. We found that most relevant posts centered around 7 major topics: Pregnant, Delivery, Pain, Breastfeeding, Water, Sleeping and Antenatal. Notably, sub-topic analysis revealed trends in repeated subject matters across these main topics, including exercise during pregnancy, breastfeeding concerns, and mental health.

In participant posts, mention of exercise was commonly associated with topics including pregnancy, delivery, pain reduction methods, and sleeping. Benefits of exercise during pregnancy for both gestational parents and neonates have been well established in the literature (23). To focus on the antenatal experiences of women, exercise has been demonstrated to improve sleep (24), lower back pain (25) and mental health (26, 27). The connections drawn in the participant posts between exercise and aspects of wellbeing during pregnancy, specifically sleep and pain management, mirror findings in the literature that suggest the role of exercise in improving these metrics. Previous studies report low rates of physical activity during pregnancy amongst women broadly in the African region, although rates vary across specific study sites (28, 29). In cross-sectional studies, women in the region have identified lack of time (30), lack of knowledge (30–33), and negative beliefs or fears about antenatal exercise as barriers (34–36). However, other studies in South Africa and Nigeria have demonstrated positive perceptions of physical activity during pregnancy and desire for increased access to information about the topic (30, 31, 37). Our findings may capitulate this trend amongst women in the region who are perceiving exercise as a beneficial practice and seeking out information to better understand its role during pregnancy.

Multiple posts in our study co-mentioned breastfeeding and delivery concerns, specifically regarding breast milk production and emergency delivery. The co-occurrence of these topics within our participants' posts demonstrates a level of awareness that these behaviors are interrelated. The World Health Organization recommends that infants should begin breastfeeding within one hour of birth and remain exclusively breastfed for at least six months, practices which have been shown to significantly decrease the risk of mortality amongst neonates (38–40). Despite these recommendations, as of 2022, only 52% of newborns are breastfed within an hour of birth and only 43% remain exclusively breastfed for 6 months after birth in Ghana (41). A recent meta-analysis of breastfeeding practices in the African region found that Caesarian section delivery was strongly associated with delayed initiation of breastfeeding, which is known to increase risk of lactogenesis failure (42, 43). Our results point to participant concern about this observed phenomenon directly, as there were ten posts with the three-word phrase “cs milk flow,” indicating a focus on milk production in relation to caesarian delivery. Additionally, “delivery emergency service” was the second most common phrase associated with the major topic of Breastfeeding, again suggesting potential understanding of or concern for how delivery method can affect breastfeeding success. Our method of extracting and analyzing the direct posts of pregnant mothers allows us glimpse at what topics from the literature are of the most interest to these women.

We also observed women's concerns around approaches to infant feeding. Posts included references to a variety of regionally and culturally familiar methods to increase breast milk supply such as palm-nut soup, kenkey and groundnut milk/mash. These remedies for augmenting milk supply are often passed down through community elders, but are also found on online Ghanaian influencer websites (44, 45). Although these practices are largely undocumented in the literature, a recent study assessing knowledge and use of traditional lactogogues by breastfeeding mothers in two regions of Ghana found that a majority of participants were aware of these methods and utilized at least one, including herb-related (e.g., dried baobab leaves), hot (e.g., hot millet porridge), or groundnut-related lactogogues (e.g., mashed kenkey with groundnut, groundnut soup) (44). Our analysis provides insight into which specific methods pregnant women in the region are considering and can help direct culturally-sensitive approaches to breastfeeding supports or education initiatives. This is especially relevant as promoting optimal breastfeeding has been deemed one of the most effective interventions to reduce early childhood deaths (46). The Enjoy Your Pregnancy group is moderated by trained and experienced midwifery professionals who provide scientifically tailored advice to the women, while also respecting their culture and traditional practices. These providers manage a delicate balance of misinformation and respect for Ghanaian cultural practices. With the recent surge in the science of complimentary and traditional medicine (47–51), offering these midwives the opportunity to participate in continuing medical education (many of which have been translated to virtual platforms since the pandemic), attend conferences such as the International Congress on Integrative Medicine and Health (52) to network with peers, and have access to open-access journals that are aligned with their professional needs, will ensure that they stay abreast of growing initiatives to integrate herbal medicines into existing healthcare infrastructures-particularly in Ghana and many other African countries (Mali, Benin, and Nigeria) (48); and advice their patients accordingly.

We found that participants are interested in understanding signs of potentially dangerous complications during pregnancy. Posts included questions regarding antenatal fundal height and monitoring pregnancy progress which can aid early antenatal detection of intrauterine growth restriction and stillbirth. The Three-Delays model is often employed as a framework to understand key barriers in the healthcare system that contribute to maternal morbidity and mortality: (1) delay in the decision to seek care; (2) delay in arrival to health facility, and (3) delay in provision of adequate care (53, 54). By engaging with the online group, the women inform their decisions to seek obstetrical care (Delay 1) by gaining a better sense of what is “normal” during pregnancy. Additionally, women warned “Don't trust the beauty” of referral hospitals, urging each other to go beyond the aesthetics of a birthing facility and consider the training, skillset and birth outcomes reported amongst providers (Delay 3). Such posts amplify the importance of the Facebook group's role in educating and empowering the women to address the first and third delays. These posts highlight the role of social media in improving maternal outcomes in regions where morbidity and mortality outcomes are so high.

Although not as prevalent in our dataset, our analysis found that mental health was discussed in posts across multiple main topics such as depression and anxiety related to pregnancy and delivery as well as depression affecting sleep. Multiple studies have identified lack of appropriate infrastructure, provider training and clinic time as barriers to the provision of adequate perinatal mental health care in Ghana (55, 56). Inadequate access to peripartum mental health care may be leading pregnant women in Ghana to turn to the internet to seek answers to their questions, gain knowledge and obtain support.

This study's findings are limited by the potential for selection bias within the sample and absence of detailed demographic or obstetric clinical information. Given that the Facebook group we analyzed is a paid subscription-based service that requires appropriate technology and internet to access, our sample may be biased toward those with higher socioeconomic statuses (SES) in the region, which may limit generalizability of the study. However, we know that technology has become increasingly ubiquitous across household income levels worldwide, so this measure may not be as reliable as an indicator of the SES of our population. Future studies should analyze data from open groups to replicate our findings. Additionally, since posters often do not frequently share specifics about their demographics or clinical information, such as age, education status, parity, gestational age, or co-morbidities, we lack knowledge of potential factors that may influence the topics about which participants are posting. Knowing the number of post-partum respondents in Figure 2 for example, would have helped the research team better contextualize participants' breastfeeding concerns after concerns in Figure 4. Diligence should be given to ensuring such clinical information is provided by users so that interventions can be properly tailored to their needs. Despite these limitations, the level of specificity regarding women's pregnancy concerns that we ascertained through innovative methodology is unique to our study. Many previous studies investigating women's use of the internet during pregnancy utilized self-reported questionnaire protocols to query what women are searching on the internet during their pregnancies (5). We analyzed the direct words of these women to tell their stories of what information they are seeking to support themselves and their pregnancies. It is incredibly important to assess the needs of pregnant women in the African region as maternal death rates remain the highest worldwide (16). Insufficient access to skilled reproductive health personnel, leading to inadequate care and education of birthing people, is cited as one factor contributing to this complex, devastating problem (16). Despite promising data supporting social media use for reproductive health promotion in higher-resourced settings (7–9), there have been limited studies investigating social media use by pregnant women in this region up until this point. To our knowledge we are the first study to apply machine learning analysis directly to posts derived from pregnant women in the West African region to better understand what types of information they are seeking or discussing online.

Though this study focused mainly on written Facebook posts, future studies could use similar methods to dissect posts from other social media platforms that incorporate visual and auditory content. Popular platforms such as TikTok and Instagram, which both allow users to upload and engage with multimedia posts, could be utilized to further examine women's interests during pregnancy and disseminate health information. For example, as mentioned, there is a desire for increased access to exercise-related information during pregnancy. A possible intervention could provide short videos on TikTok or YouTube that teach safe and simple exercises to be done at home. Other interventions could include infographic posts on commonly asked questions for birthing people (and birth partners), circulated on Instagram.

## Conclusions

In summary, our findings indicate that pregnant women in Ghana discuss common pregnancy-related topics observed across different regions, but also culturally pertinent to their context. Through our methodology, we were able to characterize distinct trends in the specific topics that women emphasized within broader categories. The benefit of such country-specific findings is that they can help to direct further needs assessments to identify actionable areas for improving patient education.

## Data Availability

The datasets presented in this study can be found in online repositories. The names of the repository/repositories and accession number(s) can be found in the article/Supplementary Material.
