# Supplementary material for: What social media analyses can tell us about Ghanaian women's concerns during pregnancy
Source: Front Digit Health. 2025 Feb 13;7:1479392. doi: 10.3389/fdgth.2025.1479392 (PMC11865226; doi:10.3389/fdgth.2025.1479392)
Supplement: Supplementary file 1 [file Datasheet1.pdf]

SUPPLEMENTARY DATA

Topic	Engagement	Video views	People reached
Wound care after cesarian section	901	840	317
How to prevent sore buttocks	771	680	386
Danger Signs in newborns	690	621	336
When to breath your baby out	686	614	333
Prevention of miscarriages	665	611	317
Fetal Distress / Meconium-stained liquor	605	536	303
How to care for your baby's skin. Advocating for your self during pregnancy	561	508	264
Pre-eclampsia and eclampsia	552	499	293
Bleeding during pregnancy and after birth	544	465	294
Proper positioning for breastfeeding / Swollen hands and feet	533	455	340
Newborns dressing and skin care	528	449	276
Hypertension in pregnancy / cord card	502	400	254
Anemia in pregnancy / preparing to breastfeed	425	342	259

How to eat during pregnancy / Twin Gestation	320	224	155
Medications during pregnancy	255	178	268
Identifying a sick child / diabetic	176	151	202
Diabetes in Pregnancy	96	70	108

Supplemental Table 1: Analysis of 'Enjoy your Pregnancy' Asynchronous Video Content Engagement, Views, and Reach. Facebook page (481 members).

Supplemental Data: Sentiment analysis of participant posts can be accessed at https://publications.midwifesally.com/sentiment.
